# Effectiveness of fluoxetine in the treatment of skin-picking

**DOI:** 10.4103/0019-5545.43065

**Published:** 2005

**Authors:** R.C. Sharma, N.L. Sharma

**Affiliations:** * Professor and Head, Department of Psychiatry, Indira Gandhi Medical College, Shimla; ** Professor and Head, Department of Dermatology, Indira Gandhi Medical College, Shimla

**Keywords:** Skin-picking, fluoxetine, neurotic excoriation

## Abstract

The case of an 18-year-old girl with skin-picking is reported. The patient used to pick at healthy skin and small skin lesions, leading to ulceration, hyperpigmentation and disfigurement. She recovered almost fully with fluoxetine. The implications of diagnosis and the need for early treatment are discussed.

## INTRODUCTION

Skin-picking, also known as neurotic excoriation or self-inflicted dermatosis, is little understood, poorly characterized and has received scant attention, especially in the psychiatric literature.^1^ However, DSM-IV-TR^2^ has listed skin-picking under the category of ‘Impulse Control Disorder Not Otherwise Specified’.

Compulsive skin-picking is a disorder characterized by excessive scratching, gouging or squeezing the normal skin, minor skin irregularities or skin lesions.^3^ Finger nails are most commonly used to excoriate the skin but teeth and instruments such as pins, nail files, tweezers or knives may also be used.^4^,^5^ Excoriations are found at multiple sites (the face being the commonest), especially on easily accessible areas of the body.^4^ About 2% of patients attending dermatology clinics have been found to suffer from compulsive skin-picking;^6^ however, the prevalence of the condition in psychiatric and non-clinical populations is unknown.^1^ The habit usually starts in adolescence, seems to be more prevalent in women than in men and follows a chronic course that is often marked by significant psychosocial distress, disfigurement and medical complications.^4^–^7^

Even after an extensive PubMed, IndMED and medIND search, the authors were unable to find any Indian study/case report, etc. on skin-picking. This case of skin-picking is reported for the rarity of the condition in the Indian literature, and to highlight the importance of early and effective treatment with fluoxetine.

## THE CASE

An 18-year-old student of standard XII, a resident of an urban area, presented to the OPD of the Department of Psychiatry, Indira Gandhi Medical College, Shimla with the complaint of skin-picking for the past 2–3 years. She used to pick at the skin around the margins of the lips and adjoining the nails, especially hangnails, often till bleeding was induced. In addition, she would scratch pimples, skin tags and other minor skin lesions. The scratched lesions would become bigger, ulcerate, and result in hypo- or hyperpigmented areas and occasionally become infected. This picking and scratching was quite frequent and was almost always associated with emotional stress or the pressure of studies, which would be relieved after skin-picking.

The patient came from a nuclear family and had one younger brother. Her father was a known case of bipolar affective disorder (maintained on prophylactic treatment from our OPD) and one of her maternal aunts was also suffering from a similar problem of skin-picking but was not on treatment. The girl had an uneventful childhood and early schooling. She was described by her mother as a shy, sensitive and introverted child with prominent obsessional traits.

Apart from the skin lesions, the general physical and systemic examinations were within normal limits. A dermatological examination revealed the presence of ulcerative areas over the tips of two fingers (left index and middle); a few, small ulcers around the lower lip; one lesion with a central papule near the left auditory meatus and another hyperpigmented healed lesion over the right cheek. The mental state examination did not reveal any significant psychiatric finding except for a strong urge to pick at the skin followed subsequently by a feeling of relief.

The patient was diagnosed as a case of compulsive skin-picking. After a session of psychoeducation, she was put on fluoxetine 20 mg/day, which was increased to 40 mg/day after two weeks as there was not much relief with the 20 mg dose. Moderate improvement was seen after four weeks and the patient was advised to continue treatment. During follow-up, the patient maintained her improvement; she was able to control herself from picking or scratching the skin except for the occasional loss of control. With time, she showed marked improvement and after another 12 weeks, fluoxetine was tapered off at the rate of 10 mg/week over a period of four weeks. The patient was counselled every time she visited the hospital in connection with her father's treatment and remained in remission. As of now, the patient has completed her postgraduation in Arts and is in remission for five years.

## DISCUSSION

Patients with compulsive skin-picking resemble those with obsessive–compulsive disorder (OCD) in terms of the repetitive, stereotyped and tension-reducing behaviour.^8^ Such patients can have features of impulse control disorders where they find themselves acting automatically.^3^ Pathological skin-picking, which most commonly presents in the context of OCD, has also been found to be frequently associated with body dysmorphic disorder, obsessive–compulsive personality disorder and borderline personality disorder.^7^ Due to these similarities, such skin-related behaviours have been reported to span a compulsivity–impulsivity continuum from the purely obsessive–compulsive to the purely impulsive, with mixed symptoms in between.^9^

Depression, anxiety and mood disorders are reported to be common co-morbid psychiatric diagnoses in patients with psychogenic excoriation.^4^,^5^ This patient did not have any associated psychiatric co-morbidity; however, two important findings that deserve mention are (i) the prominent obsessional traits in the patient, and (ii) the presence of bipolar affective illness in her father and skin-picking in the maternal aunt. Both these associations suggest that skin-picking should be included in the family of OCD spectrum disorders which, in turn, belong to the larger family of affective spectrum disorders, a hypothesis put forward by McElroy and colleagues.^9^ The positive response to fluoxetine in this case corroborates a similar finding by other authors.^5^,^8^,^10^

This case, which has been followed up for more than five years now, highlights the effectiveness of fluoxetine in the treatment of skin-picking and the need for early institution of treatment, which would not only curtail the period of suffering but ensure long-lasting improvement in such cases.
